# Assessment of knowledge, attitudes, and practices regarding menstruation and menstrual hygiene among early-reproductive aged women in Bangladesh: a cross-sectional survey

**DOI:** 10.3389/fpubh.2023.1238290

**Published:** 2023-11-28

**Authors:** Abu Bakkar Siddique, Sudipto Deb Nath, Mahfuza Mubarak, Amena Akter, Sanjida Mehrin, Mst Jemi Hkatun, Antara Parvine Liza, M. Ziaul Amin

**Affiliations:** ^1^Department of Public Health and Informatics, Jahangirnagar University, Savar, Dhaka, Bangladesh; ^2^Centre for Advanced Research Excellence in Public Health, Savar, Dhaka, Bangladesh; ^3^International Centre for Research, Innovation, Training and Development (ICRITD), Dhaka, Bangladesh; ^4^Army Medical College Jashore, Jashore, Bangladesh; ^5^Department of Genetic Engineering and Biotechnology, Jashore University of Science and Technology, Jashore, Bangladesh; ^6^Department of Agriculture, Bangabandhu Sheikh Mujibur Rahman Science and Technology University, Gopalganj, Dhaka, Bangladesh; ^7^Department of Political Studies, Shahjalal University of Science and Technology, Sylhet, Bangladesh; ^8^Shahjalal University of Science and Technology, Sylhet, Bangladesh

**Keywords:** menstruation, menstrual hygiene, knowledge, attitudes, practices, health effect, Bangladesh, early-reproductive age

## Abstract

**Background:**

Poor menstrual hygiene practices during menstruation increases the risk of reproductive tract infections, absenteeism, and negative impact on school performance. Despite being a global problem, there is a lack of knowledge and misconceptions about menstrual hygiene among women of all ages, especially in developing countries like Bangladesh. The study aims to assess the knowledge, attitudes, and practices toward menstrual hygiene among early reproductive-aged women in Bangladesh to help policymakers and planners take effective initiatives.

**Methodology:**

A cross-sectional survey was conducted between July and December 2022 in Dhaka, Rajshahi, Chittagong, Sylhet, and Barisal regions of Bangladesh. A convenience sampling technique was utilized to recruit a total of 1,214 participants (100% female; mean age: 22.87 ± 2.64 years). A semi-structured questionnaire including informed consent, socio-demographic information, as well as questions regarding knowledge (6-item), attitudes (7-item), and practices (6-item) toward menstruation and menstrual hygiene practices, was used to conduct the survey. All analyses were performed using the STATA (Version 15.0) and Statistical Package for the Social Sciences (SPSS version 25.0).

**Result:**

The mean scores of the knowledge, attitudes, and practices were 4.9 ± 1.51 (out of 6), 12.58 ± 1.58 (out of 14), and 8.80 ± 1.87 (out of 12), respectively. The higher scores of knowledge, attitudes, and practices were significantly associated with several socio-demographic and menstruation-related factors (education, family type, type of menstruation, duration of menstruation, etc.).

**Conclusion:**

This study identified several key factors associated with improved knowledge, attitudes, and practices related to menstrual hygiene, including higher education levels, student status, younger age, non-alcohol consumption, and regular menstrual cycles. To enhance menstrual hygiene practices among women, it is crucial to implement targeted interventions that address knowledge disparities and tackle social and environmental influences.

## 1 Introduction

Menstruation, often known as a period, is regular vaginal bleeding brought on by the monthly release of blood and tissue from the uterine lining (1). The beginning of menstruation, or menarche (puberty), together with the mental, emotional, and social changes up until the end of reproductive age, or menopause (the cessation of menstruation), are the most significant physiological transitional processes in women. Menstrual hygiene (MH) is a hygienic practices that takes place during menstruation. Proper menstrual hygiene involves thoroughly washing the external genitalia, utilizing sanitary napkins, tampons, menstrual cups, and similar products (2), while also replacing the napkin every 3 to 4 h to prevent odor (3).

Indian investigations discovered a connection between MH practices and urogenital infection (4, 5). According to a study on adolescent women in Ethiopia, stress-related mental states, such as low confidence and self-esteem, as well as academic failure owing to absence, all enhance the chance of infection (6). Another study of adolescent women from Rwanda and Ethiopia discovered that poor MH was also associated with infertility, reproductive tract infections (RTI), anemia, vaginal discharge with odor, itching, and other conditions (7). A study in Bangladesh of 2,332 schoolgirls, aged 11 to 17, from 700 schools indicated that menstruation had a detrimental impact on 32% of the participants’ academic performance and that 41% of them missed an average of 2.8 days per cycle (8). In addition, many women lack access to basic sanitary facilities (9), which makes it challenging for them to manage their MH owing to lack of facilities and proper knowledge (10). Unsanitary activities during menstruation increase the risk of infection or gynecological disorders in women. The frequency of reproductive tract infections is three times higher in women who practices poor MH (1). Menstrual absorbents may include these viruses and bacteria, therefore improper disposal of them could result in the transmission of hepatitis and HIV (11). Although the majority of the research focuses on young girls in school and adolescence, MH is a global issue that affects women and girls of all ages. Not just young girls, but women of many ages struggle with inadequate MH. Women frequently lack access to necessities like sanitary products, potable water, and private restrooms. Major health problems like infections and the spread of diseases may come from this (10, 12). Bangladesh and other lower-income nations are therefore very concerned about MH-related knowledge and practices.

Factors contributing to educational institutions and workplace absences among women included their attitudes, limited knowledge, and misconceptions about menstruation, as well as family restrictions, and inadequate facilities at the workplace (6). In Bangladesh as well as many developing countries, MH management among reproductive aged women remains poorly discussed so far. A pilot study on rural and urban women in Bangladesh found that they had got a chance to submit any question, 45% of them wanted to know about menstruation and 35% of these questions were about experiences of menstrual bleeding (13). So, there is a huge knowledge gap regarding MH and a proper KAP study can address this knowledge gap. Achieving SDG’s goal of sustainable water and sanitation, good health, and gender equality can be accelerated by assessing MH safely. The effective and timely provision of accurate information about menstruation and its management within supportive social environments that are free from menstrual stigma, as implied by this study, can enhance women’s capacity to manage menstrual hygiene (14).

Moreover, early-reproductive age is a critical period for women’s reproductive health and poor MH during this period may be indicative of various infections or other health conditions (15). There have been numerous studies on Bangladeshi schoolchildren and teenagers, but none have looked specifically at how early-reproductive-aged women in Bangladesh feel and know about MH. This study evaluates the knowledge, attitudes, and practices of early reproductive women concerning MH, which will assist policymakers and planners in making successful decisions by taking into account the needs of women today. The study’s conclusions will be useful in preventing menstrual disorders, infections, and difficulties associated with menstruation, as well as in promoting menstrual health by altering lifestyle and quality of life.

## 2 Materials and methods

### 2.1 Study area

The study was carried out in different parts of Bangladesh by self-reported questionnaire-based cross-sectional survey. Data was collected from Dhaka, Rajshahi, Chittagong, Sylhet and Barisal regions of Bangladesh between July and December 2022.

### 2.2 Sample size

The sample size was calculated using the following equation:


$$n=\frac{z^{2}pq}{d^{2}};n=\frac{1.96^{2}\times 0.5\times \left(1-0.5\right)}{0.05^{2}}=384.16\approx 384$$

Here,

*n* = number of samples.

*z* = 1.96 (95% confidence level).

*p* = prevalence estimate (50% or 0.5) (as no study found).

*q* = (1-*p*).

*d* = precession of the prevalence estimate (10% of 0.05).

We expected that the current study’s prevalence estimate (p) would be 50%. A sample size of 423.5 ≈ 424 people was estimated based on a 10% non-response rate. This estimate was exceeded by our sample size. However, 1,214 participants were recruited to ensure the strength of the study.

### 2.3 Study design, participants, and procedure

The current study utilized a cross-sectional survey design based on self-reported questionnaires, conducted between July and December 2022. The participants were enrolled using a non-probability sampling (convenient sampling) technique (16). Each participant took approximately 10–15 min to complete the interview. Initially, 1,310 participants attended the surveys. After removing incomplete responses, the final analysis included 1,214 surveys. The data were gathered using a paper-based semi-structured questionnaire written in Bangla (the participant’s native language) from house to house. As MH is a very sensitive issue, the data was collected only by female research assistants and strict confidentiality was maintained.

A pilot test was carried out with 10 participants from the same population (target group) to determine the acceptability and transparency of the questionnaire. Following the pilot testing, a few minor adjustments were incorporated into the questionnaire. These data were not included in the final analysis. The first page of the questionnaire had an informed consent statement attached to it that explained the study’s objectives, procedures, and the participant’s right to decline participation. Before starting the survey, “participants were asked to provide informed consent (i.e., *“Are you willing to participate in this study voluntarily and spontaneously?”*). The inclusion criteria of the participants included: (i) women at early reproductive age (18 to 35 years of age) (17), (ii) experienced menstruation at their reproductive age, and (iii) living in Bangladesh. The participants below 18 years and over 35 years were excluded at the time of the interview.

### 2.4 Measures

#### 2.4.1 Socio-demographic measures and determinants of menstruation and MH

Socio-demographic information was gathered by questions about age (later categorized as 18–24 years and 25–35 years), marital status (married/unmarried), education (below university/ university level), occupation (student/unemployed/employed/others), place of residence (Rural/Urban), family category (up to 4 members/more than 4 members), monthly family income (less than 15,000 Bangladeshi Taka [BDT]/1500BDT to 30,000 BDT/more than 30,000 BDT), Having children (yes/no), (OCP) oral contraceptive usage (yes/no).

#### 2.4.2 Health-related measures and determinants of MH

Body Mass Index [BMI] (underweight/normal/overweight) [height and weight were measured by scales and then BMI was calculated], daily sleeping time (Less than 7 h/7 to 9 h/More than 9 h), smoking status (yes/no), alcohol intake (yes/no), social media usage in a day (less than 2 h/2 to 5 h/more than 5 h).

#### 2.4.3 Menstruation and MH-related measures

Menstruation starting age (8 to 11 years/12 to 14 years/more than 14 years), type of menstruation (regular/irregular), the average duration of menstruation (less than 3 days/3 to 6 days/7 or more days).

#### 2.4.4 Knowledge, attitudes, and practices measures

A total of 19 questions regarding knowledge (6-item), attitudes (7-item), and practices (6-item) toward MH, were used in the present study which were adopted from previous literature through extensive literature review (3, 18–20).

**Figure 1:**
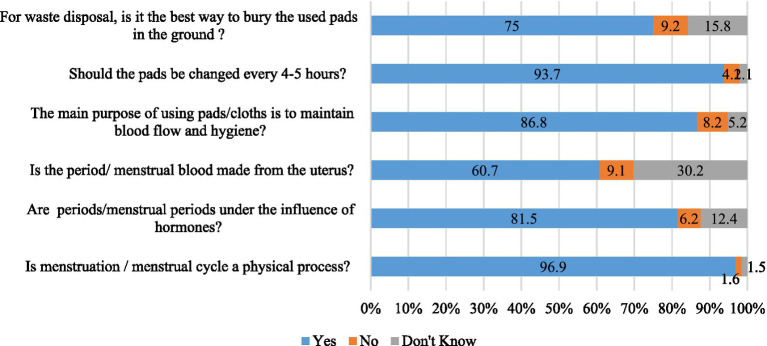
Knowledge regarding MH and menstruation.

Six-item questions with three options (e.g., yes/no/do not know) related to knowledge regarding MH and its health effects were asked to the participants (e.g., *“Is menstruation/menstrual cycle a physical process?,” “Are menstruations/menstrual cycle under the influence of hormones”*) (see details in Figure 1). During analysis, “yes” responses were coded as “1”; whereas “no” and “do not know” responses were coded as “0.” The total score was obtained by summating the scores of all items and ranges from 0–6, with a higher score indicating a higher level of knowledge. In addition, sources of knowledge regarding menstruation/period were also recorded from the participants.

**Figure 2:**
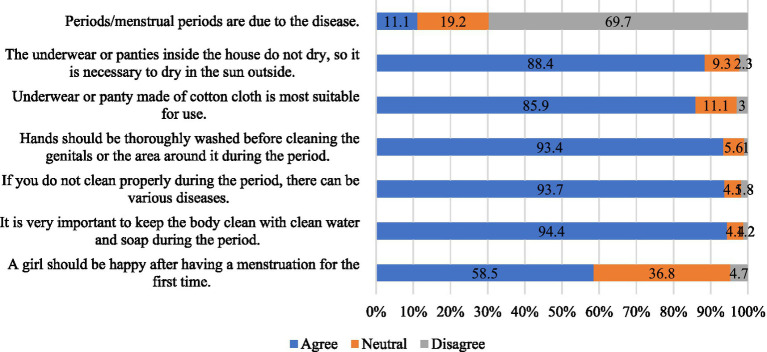
Attitude regarding MH and menstruation.

To assess the attitudes toward MH, seven questions were used with a three-point Likert scale (e.g., 0 = disagree, 1 = neutral, 2 = agree). Examples of such questions include: *“A girl should be happy after having a menstruation for the first time.,” “It is very important to keep the body clean with clean water and soap during the menstruation”* (see details in Figure 2). The total score was obtained by summating the scores of all items and ranges from 0–14, with the higher score indicating a greater level of positive attitudes.

**Figure 3:**
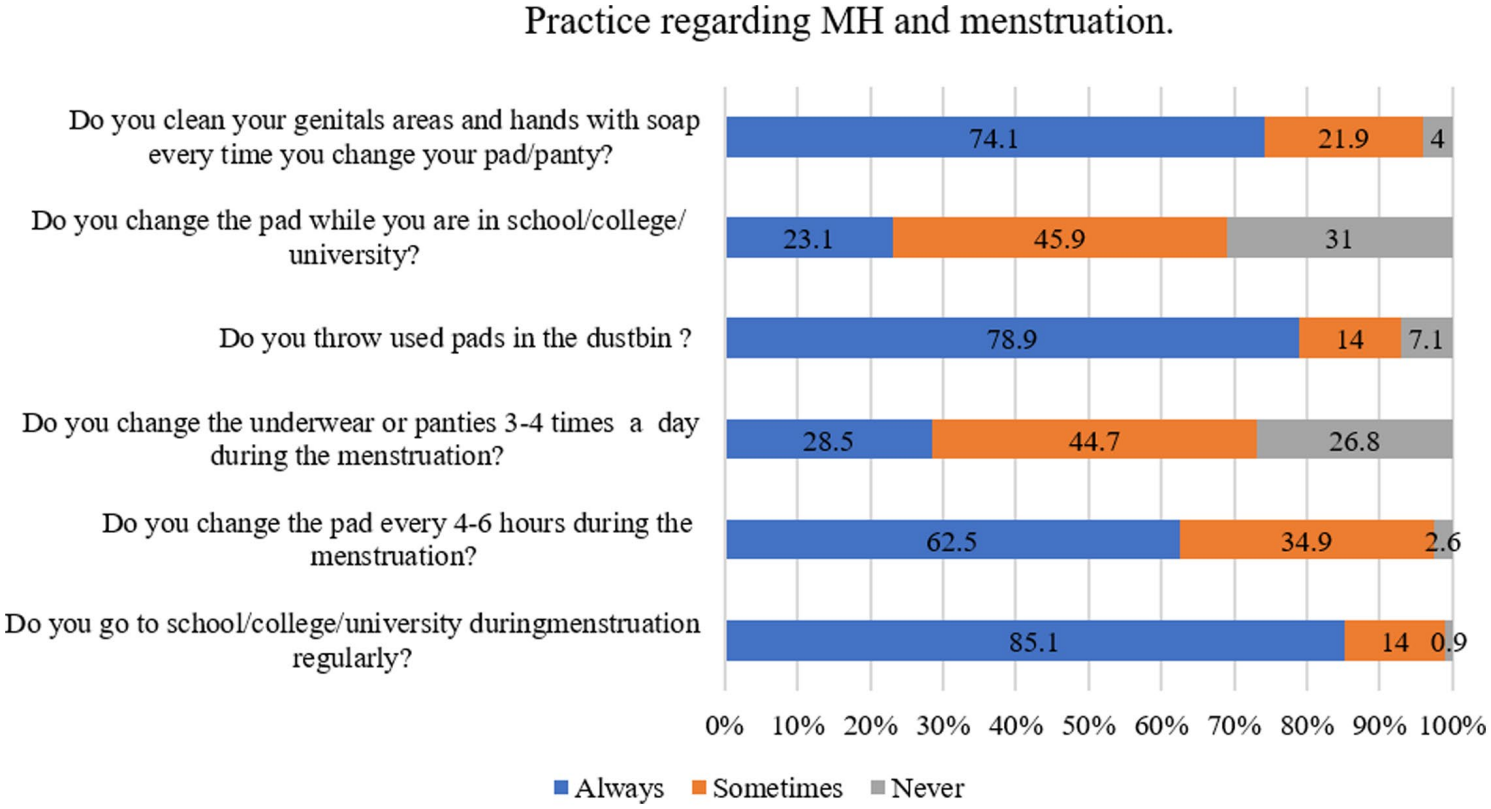
Practice regarding MH and menstruation.

To document the practices status, the participants were asked six questions (e.g., “Do you go to school/college/university during menstruation regularly?,” “Do you change the pad every 4–6 h during the menstruation?,” “Do you change the underwear or panties 3–4 times a day during the menstruation?”) (see details in Figure 3) with three possible responses (e.g., never, sometimes, always). During analysis, “never” responses were coded as “0,” “sometimes” responses were coded as “1,” and “always” were coded as “2.” The total score was obtained by summating the scores of all items and ranges from 0–12, with a higher score indicating a higher level of practices.

### 2.5 Statistical analysis

The data were analyzed using Statistical Package for Microsoft Excel (version 2021), Social Sciences (SPSS version 25.0), and STATA (version 15.0). Cleaning, coding, and sorting were performed using the help of Microsoft Excel. Then, the Excel file was imported in the SPSS software and the descriptive statistics (i.e., frequencies, percentages, means, and standard deviations) were computed. Finally, bivariate and multivariable linear regression analyses were performed using the STATA including the total scores of knowledge, attitudes, and practices measures. A value of *p* less than 0.05 was regarded as significant for all of the analyses.

### 2.6 Ethics statement

The survey was carried out in accordance with the Helsinki Declaration of 1975. The Ethics Review Committee of the Faculty of Biological Science and Technology, Jessore University of Science and Technology, Jessore-7408, Bangladesh examined and approved the study protocol [Ref: ERC/FB ST/JUST/2022-l -0 L]. All respondents were informed of the study’s goal, procedure, and ability to withdraw their data. Prior to completing the study, each participant provided informed consent. Participants were advised that all of their information would be kept anonymous and confidential.

## 3 Results

### 3.1 General characteristics of the participants

A total of 1,214 participants were included in the final analysis. Of them, the majority were aged between 18 to 24 years (87.4%), unmarried (83.9%), had a monthly family income of more than 30,000 BDT (45.9%), and were students (87%). 91.4% had a university level of education, 79.2% were from nuclear families and 82.2% were from urban areas. Most women did not have any children (94.23%), and did not use any oral contraceptive (94.0%). 96.2% of women had normal BMI and 59.80% of women had an average daily sleeping time of 7 to 9 h. The majority reported that they did not smoke (96.8%) and did not take alcohol 97.69%. The proportion of the participants who reported using social media 2 to 5 h in a day was 58.2%. 75% of women reported that their menstrual cycle began between the ages of 12 and 14. The majority of women (76.7%) have regular menstruation, and 75.1% of them claimed their menstruation lasted between three and six days (Table 1).

**Table 1:** General characteristics of the population.

Socioeconomic information	
Age	
18 to 24 years	1,061 (87.4)
25 to 25 years	153 (12.6)
Marital status	
Unmarried	1,018 (83.9)
Married	196 (16.1)
Education level	
Below university	105 (8.6)
University level	1,109 (91.04)
Monthly family income	
<15,000 BDT	242 (19.9)
15,000–30,000 BDT	415 (34.2)
> 30,000 BDT	557 (45.9)
Occupation	
Student	1,058 (87)
Unemployed	34 (2.8)
Employed	63 (5.2)
Others	59 (5)
Place of residence	
Rural	216 (17.8)
Urban	998 (82.2)
Family type	
Nuclear	961 (79.2)
Large	253 (20.8)
Children	
Yes	70 (5.77)
No	1,144 (94.23)
OCP usage	
Yes	73 (6.0)
No	1,141 (94.0)
Basic health related information	
BMI	
Underweight	192 (15.8)
Normal	840 (96.2)
Overweight	182 (15.2)
Daily sleeping status	
Less than 7 h	392 (32.29)
7 to 9 h	726 (59.80)
More than 9 h	96 (7.91)
Smoking	
Yes	39 (3.2)
No	1,175 (96.8)
Alcohol intake	
Yes	28 (2.31)
No	1,186 (97.69)
Social media usage in a day	
Less than 2 h	271 (22.3)
2 to 5 h	706 (58.2)
More than 5 h	237 (19.5)
Menstruation/Period related information	
Menstruation starting age	
8 to 11 years	199 (16.4)
12 to 14 years	910 (75.0)
More than 14 years	105 (8.6)
Type of menstruation	
Regular	931 (76.7)
Irregular	283 (23.3)
Duration of menstruation	
Less than 3 days	138 (11.4)
3 to 6 days	912 (75.1)
7 or more days	164 (13.5)

Note: BDT Bangladeshi Taka, 1 BDT 0.0091 U$$ in 4 November, 2023.

### 3.2 Knowledge regarding menstruation and MH

**Figure 4:**
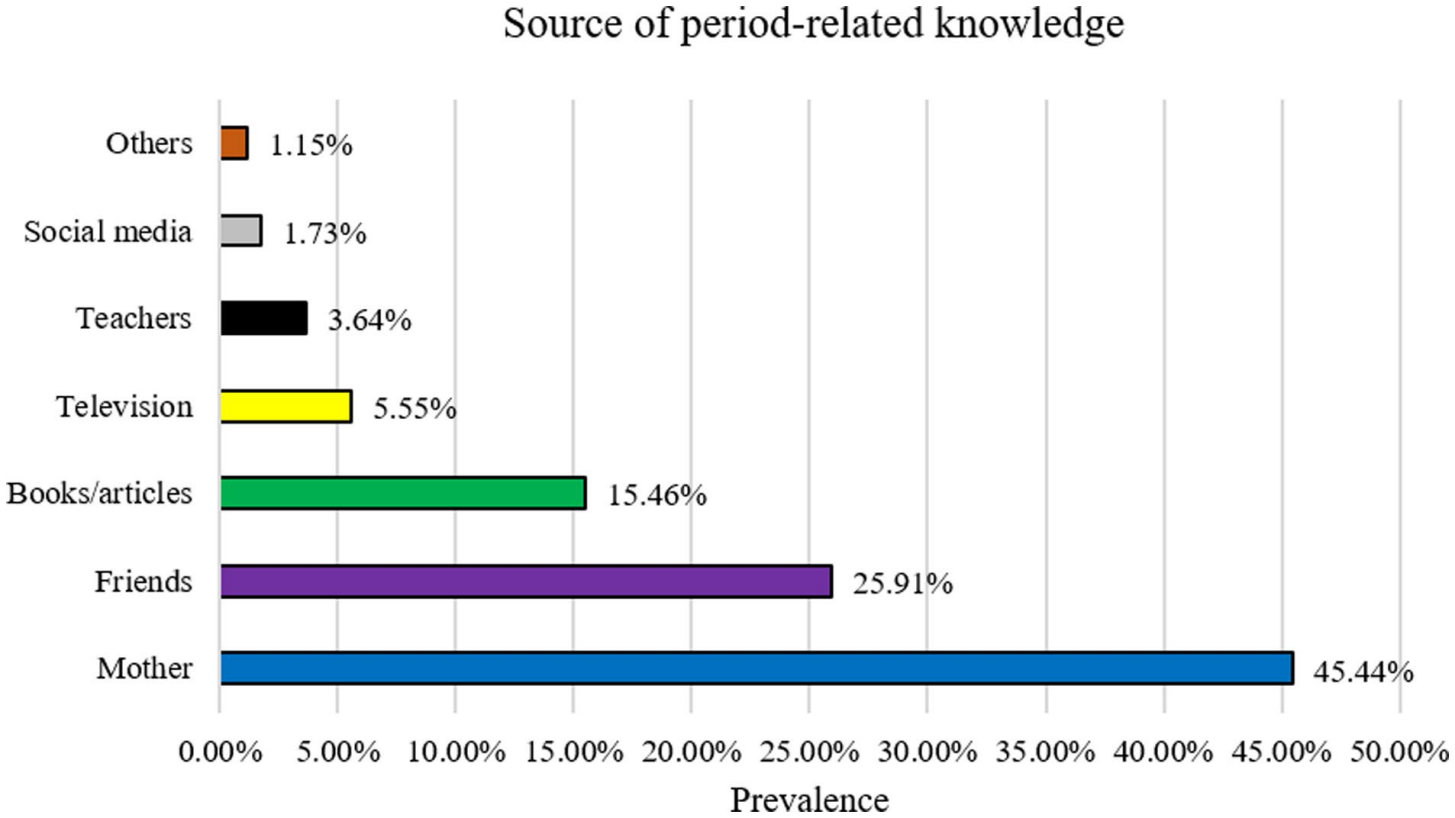
Source of period-related knowledge.

The mean score of the knowledge items was 4.9 (SD = 1.51) out of 6, indicating an overall correct percentage of 81.66%. As per as multiple linear regression analysis, the positively predicting factors of knowledge score included: (i) having education at the university level (*ꞵ* = 0.12, *p* < 0.024) in reference to ‘below university’, (ii) being a student (*ꞵ* = 0.14, *p* = 0.013) in reference to ‘other occupation’, (iii) living with a nuclear/small family (*ꞵ* = 0.08, *p* = 0.003) in reference to ‘large’, (iv) having daily sleeping time of less than 7 h (*ꞵ* = 0.13, *p* = 0.017) in reference to ‘more than 9 h’, and (v) not taking alcohol (*ꞵ* = 0.06, *p* = 0.023) in reference to ‘yes’ (Table 2). Figure 4 demonstrates the sources of knowledge regarding menstruation and MH.

**Table 2:** Regression analysis predicting knowledge.

Variables	Overall	Bivariable regression analysis					Multivariable regression analysis				
	Mean (SD)	*B*	SE	*t*	*ꞵ*	Value of *p*	*B*	SE	*t*	*ꞵ*	Value of *p*
Age											
18 to 24 years	5.00 (1.11)	0.43	0.09	4.37	0.12	**<0.001**	0.03	0.12	0.25	<0.01	0.803
25 to 35 years	4.57 (1.38)	Ref.					Ref.				
Marital status											
Unmarried	4.99 (1.11)	0.25	0.09	2.83	0.08	**0.005**	−0.09	0.11	−0.82	−0.02	0.412
Married	4.73 (1.35)	Ref.					Ref.				
Education level											
Below university	4.51 (1.28)	Ref.					Ref.				
University	4.99 (1.14)	0.47	0.11	4.04	0.11	**<0.001**	0.27	0.12	2.26	0.06	**0.024**
Monthly family income											
> 15,000 BDT	5.00 (1.11)	0.06	0.08	0.76	0.02	0.448	**-----**	**-----**	**-----**	**-----**	**------**
15,000–30,000 BDT	4.95 (1.22)	0.01	0.07	0.25	0.00	0.802	**-----**	**-----**	**------**	**-----**	**------**
> 30,000 BDT	4.93 (1.13)	Ref.									
Occupation											
Student	5.03(1.09)	0.83	0.15	5.51	0.24	**<0.001**	0.49	0.19	2.48	0.14	**0.013**
Unemployed	4.71 (1.45)	0.51	0.24	2.12	0.07	**0.034**	0.39	0.25	1.54	0.05	0.124
Employed	4.49 (1.38)	0.30	0.20	1.48	0.05	0.139	0.05	0.21	0.24	0.01	0.867
Others	4.19 (1.47)	Ref.					Ref.				
Place of residence											
Rural	4.92 (1.13)	0.03	0.08	−0.44	−0.01	0.660	**-----**	**----**	**-----**	**-----**	**------**
Urban	4.95 (1.38)	Ref.									
Family type											
Nuclear	5.03 (1.09)	0. 38	0.08	4.80	0.13	**<0.001**	0.25	0.08	2.99	0.08	**0.003**
Large	4.64 (1.35)	Ref.					Ref.				
Children											
Yes	4.33 (1.41)	Ref.					Ref.				
No	4.99 (1.13)	0. 65	0.14	4.65	0.13	**<0.001**	0.19	0.18	1.08	0.03	0.281
OCP usage											
Yes	4.53 (1.43)	Ref.					Ref.				
No	4.97 (1.13)	0. 44	0.13	3.16	0.09	**0.002**	−0.02	0.16	−0.20	−0.01	0.990
BMI											
Underweight	5.17 (0.96)	0.34	0.11	2.87	0.10	**0.004**	0.21	0.11	1.82	0.06	0.069
Normal	4.92 (1.18)	0. 09	0.09	0.99	0.03	0.325	0.03	0.09	0.33	0.01	0.743
Overweight	4.83 (1.20)	Ref.					Ref.				
Daily sleeping time											
Less than 7 h	5.02 (1.08)	0.36	0.13	2.77	0.14	**0.006**	0.31	0.13	2.39	0.12	**0.017**
7 to 9 h	4.95 (1.14)	0.29	0.12	2.32	0.12	**0.020**	0.23	0.12	1.84	0.09	0.066
More than 9 h	4.66 (1.53)	Ref.					Ref				
Smoking status											
Yes	4.82 (1.95)	Ref.									
No	4.82 (1.23)	0.13	0.18	0.70	0.02	0.484	**-----**	**-----**	**-----**	**-------**	**------**
Alcohol intake											
Yes	4.25 (1.27)	Ref.					Ref.				
No	4.96 (1.16)	0.71	0.22	3.24	0.09	**0.001**	0.50	0.22	2.27	0.06	**0.023**
Social media usage in a day											
Less than 2 h	4.82 (1.14)	Ref.					Ref.				
2 to 5 h	5.00 (1.15)	0.18	0.08	2.20	0.07	**0.028**	0.32	0.15	2.38	0.06	0.057
More than 5 h	4.95 (1.20)	0.13	0.10	1.34	0.04	0.180	0.25	0.13	1.86	0.06	0.066
Menstruation starting age											
8 to 11 years of age	4.78 (1.11)	Ref.									
12 to 14 years of age	5.00 (1.14)	0.21	0.09	2.35	0.07	**0.019**	0.19	0.08	2.18	0.07	0.059
More than 14 years of age	4.84 (1.32)	0.05	0.13	0.39	0.01	0.698	0.10	0.13	0.79	0.02	0.432
Type of menstruation											
Regular	4.97 (1.15)	0.11	0.07	1.43	0.04	0.154	**------**	**----**	**-----**	**-----**	**--------**
Irregular	4.86 (1.19)	Ref.									
Duration of menstruation											
Less than 3 days	4.75 (1.13)	Ref.					Ref.				
3 to 6 days	4.98 (1.14)	0.23	0.10	2.21	0.08	**0.027**	0.08	0.10	0.84	0.03	0.399
More than 6 days	4.94 (1.26)	0.19	0.13	1.44	0.05	0.149	0.12	0.13	0.91	0.03	0.265

Note: B = unstandardized regression coefficient; SE = Standard error; *β* = standardized regression coefficient; Bold indicates significant (*p* < 0.05).

### 3.3 Attitudes regarding menstruation and MH

The mean score of the attitudes items was 12.58 (SD = 1.57) out of 14, indicating an overall correct percentage of 89.86%. As per as multiple linear regression analysis, the positively predicting factors of attitudes score included: (i) having age between 18 to 24 years (*ꞵ* = 0.16, *p* < 0.001) in reference to ‘25 to 35 years’, (ii) being a student (*ꞵ* = 0.13, *p* = 0.020) in reference to ‘other occupation’, (iii) living with a nuclear/small family (*ꞵ* = 0.10, *p* = 0.003) in reference to ‘large’, (iv) smoker (*ꞵ* = 0.05, *p* = 0.035) in reference to ‘non-smoker’, (v) not taking alcohol (*ꞵ* = 0.05, *p* = 0.035) in reference to ‘yes’, and (vi) having regular type of menstruation (*ꞵ* = 0.05, *p* = 0.002) in reference to ‘irregular’ (Table 3).

**Table 3:** Regression analysis predicting attitude.

Variables	Overall	Bivariate regression analysis					Multivariable regression analysis				
	Mean (SD)	*B*	SE	*t*	*ꞵ*	Value of *p*	*B*	SE	*t*	*ꞵ*	Value of *p*
Age											
18 to 24 years	12.74 (1.39)	1.19	0.13	9.08	0.25	**<0.001**	0.59	0.16	3.6 0	0.12	**<0.001**
25 to 35 years	11.54 (2.25)	Ref.					Ref.				
Marital status											
Unmarried	12.68 (1.41)	0.57	0.12	4.76	0.08	**<0.001**	−0.13	0.12	−1.12	−0.03	0.315
Married	12.10 (2.18)	Ref.					Ref.				
Education											
Below university	11.98 (1.98)	Ref.					Ref.				
University	12.65 (1.52)	0.66	0.15	4.16	0.11	**<0.001**	0.29	0.15	1.87	1.87	0.062
Monthly family income											
> 15,000 BDT	12.71 (1.53)	0.10	0.12	0.90	0.02	0.367	**------**	**----**	**-----**	**-----**	**-----**
15,000–30,000 BDT	12.50 (1.69)	−0.10	0.10	−1.01	−0.03	0.315	**-----**	**-----**	**-----**	**------**	**-----**
> 30,000 BDT	12.60 (1.50)	Ref.									
Occupation											
Student	12.74 (1.38)	1.60	0.20	7.89	0.34	**<0.001**	0.61	0.26	2.33	0.13	**0.020**
Unemployed	11.82 (1.98)	0.68	0.32	2.10	0.07	**0.036**	0.28	0.33	0.87	0.03	0.386
Employed	11.79 (1.96)	0.65	0.27	2.39	0.01	**0.017**	0.16	0.28	0.59	0.02	0.558
Others	11.14 (2.69)	Ref.					Ref.				
Place of residence											
Rural	12.53 (1.63)	Ref.									
Urban	12.60 (1.56)	0.06	0.11	0.57	0.01	0.566	**----**	**----**	**-----**	**-----**	**------**
Family type											
Nuclear	12.71 (1.40)	0.06	0.10	5.53	0. 15	**<0.001**	0.32	0.10	3.02	0.08	**0.003**
Large	12.11 (2.04)	Ref.					Ref.				
Children											
Yes	11.30 (2.54)	Ref.					Ref.				
No	12.67 (1.46)	1.36	0.18	7.20	0.20	**<0.001**	0.26	0.22	1.22	0.04	0.311
OCP usage											
Yes	11.56 (2.39)	Ref.					Ref.				
No	12.65 (1.48)	1.09	0.18	5.82	0.16	**<0.001**	0.26	0.22	1.22	0.04	0.222
BMI											
Underweight	12.90 (1.26)	0.38	0.16	2.37	0.08	**0.018**	0.08	0.15	0.57	0.02	0.570
Normal	12.53 (1.57)	0.01	0.12	0.12	0.90	0.903	−0.14	0.12	−1.14	−0.04	0.254
Overweight	12.52 (1.83)	Ref.									
Daily sleeping status											
Less than 7 h	12.64 (1.51)	0.45	0.17	2.56	0. 14	**0.011**	0.32	0.17	1.93	0.09	0.054
7 to 9 h	12.62 (1.51)	0.43	0.17	2.58	0.12	**0.010**	0.28	0.16	1.75	0.08	0.080
More than 9 h	12.18 (2.16)	Ref.					Ref.				
Smoking status											
Yes	11.36 (2.02)	Ref.					Ref.				
No	12.63 (1.54)	1.26	0.25	5.01	0.14	**<0.001**	0.55	0.26	2.11	0.05	**0.035**
Alcohol intake											
Yes	10.75 (2.68)	Ref.					Ref.				
No	12.63 (1.51)	1.88	0.29	6.35	0.17	**<0.001**	1.12	0.31	3.58	0 0.10	**<0.001**
Social media usage in a day											
Less than 2 h	12.51 (1.63)	Ref.									
2 to 5 h	12.62 (1.59)	0.10	0.11	0.96	0.03	0.336	**-----**	**-----**	**-----**	**-----**	**-----**
More than 5 h	12.59 (1.46)	0.08	0.14	0.58	0.02	0.336	**-----**	**-----**	**-----**	**-----**	**-----**
Menstruation starting age											
8 to 11 years of age	12.40 (1.74)	Ref.									
12 to 14 years of age	12.66 (1.50)	0.26	0.12	2.13	0.07	**0.033**	0.21	0.11	1.86	0.05	0.063
More than 14 years of age	12.29 (1.82)	−0.11	0.18	−0.61	−0.02	0.539	−0.01	0.18	−0.05	−0.01	0.958
Type of menstruation											
Regular	12.69 (1.49)	0.42	0.10	3.97	0.11	**<0.001**	0.21	0.10	2.04	0.05	**0.041**
Irregular	12.27 (1.80)	Ref.									
Duration of menstruation											
Less than 3 days	12.51 (1.80)	Ref.					Ref.				
3 to 6 days	12.61 (1.52)	0.10	0.14	0.73	0.02	0.467	**-----**	**----**	**-----**	**------**	**-------**
More than 6 days	12.52 (1.77)	0.07	0.18	0.09	<0.01	0.925	**-----**	**----**	**-----**	**------**	**-------**

Note: B = unstandardized regression coefficient; SE = Standard error; *β* = standardized regression coefficient; Bold indicates significant (*p* < 0.05).

### 3.4 Practices regarding menstruation and MH

The mean score of the practices items was 8.80 (SD = 1.87) out of 12, indicating an overall correct percentage of 73.33%.As per as multiple linear regression analysis, the positively predicting factors of attitudes score included: (i) having age between 25 to 35 years (*ꞵ* = 0.06, *p* < 0.035) in reference to ‘ss18 to 24 years’, (ii) having education at the university level (*ꞵ* = 0.07, *p* = 0.013) in reference to ‘below university’, (iii) living in an urban area (*ꞵ* = 0.05, *p* = 0.035) in reference to ‘rural’, (iv) living with a large family (*ꞵ* = 0.07, *p* = 0.010) in reference to ‘nuclear’, (v) having duration of menstruation between 3 to 6 days (*ꞵ* = 0.07, *p* = 0.032) in reference to ‘more than 6 days’ (Table 4).

**Table 4:** Regression analysis predicting practice.

Variables	Overall	Bivariate regression analysis					Multivariable regression analysis				
	Mean (SD)	*B*	SE	*t*	*ꞵ*	Value of *p*	*B*	SE	*t*	*ꞵ*	Value of *p*
Age											
18 to 24 years	8.75 (1.88)	Ref.					Ref.				
25 to 35 years	9.14 (1.84)	0.38	0.16	2.38	0.06	**0.018**	0.34	0.16	2.11	0.06	**0.035**
Marital status											
Unmarried	8.78 (1.89)	Ref.									
Married	8.90 (1.83)	0.11	0.14	0.79	0.02	0.428	**----**	**----**	**-----**	**-----**	**-----**
Education											
Below university	8.43 (1.98)	Ref.					Ref.				
University	8.84 (1.86)	0.40	0.19	2.13	0.06	**0.034**	0.47	0.13	2.11	0.07	**0.013**
Monthly family income											
> 15,000 BDT	8.67 (1.82)	Ref.									
15,000–30,000 BDT	8.73 (1.89)	0. 05	0.15	0.36	0.01	0.721	**----**	**----**	**-----**	**-----**	**-----**
> 30,000 BDT	8.91 (1.89)	0.23	0.14	1.64	0.06	0.102	**-----**	**----**	**-----**	**-----**	**-----**
Occupation											
Student	8.76 (1.87)	−0.15	0.25	−0.61	−0.02	0.544	**----**	**----**	**-----**	**-----**	**-----**
Unemployed	9.06 (1.94)	0.14	0.40	0.36	0.01	0.722	**-----**	**----**	**-----**	**-----**	**-----**
Employed	9.19 (1.94)	0.27	0.33	0.81	0.03	0.418	**----**	**----**	**-----**	**-----**	**-----**
Others	8.92 (1.93)	Ref.									
Place of residence											
Rural	8.57 (1.90)	Ref.					Ref.				
Urban	8.85 (1.87)	0.28	0.14	2.00	0.05	**0.046**	0.29	0.13	2.11	0.05	**0.035**
Family type											
Nuclear	8.73 (1.90)	Ref.					Ref.				
Large	9.07 (1.76)	0.33	0.13	2.54	0.07	**0.011**	0.32	0.13	2.42	0.07	**0.010**
Children											
Yes	8.99 (1.87)	0.19	0.23	0.85	0. 20	0.396	**----**	**----**	**-----**	**-----**	**-----**
No	8.79 (1.88)	Ref.									
OCP usage											
Yes	8.92 (1.82)	0.12	0.22	0.55	0. 01	0.583	**----**	**----**	**-----**	**-----**	**-----**
No	8.79 (1.88)	Ref.									
BMI											
Underweight	8.71 (2.01)										
Normal	8.82 (1.87)	0.10	0.15	0.73	0.08	0.466	**----**	**----**	**-----**	**-----**	**-----**
Overweight	8.82 (1.77)	0.11	0.19	0.57	0.90	0.570	**-----**	**----**	**-----**	**-----**	**-----**
Daily sleeping status											
Less than 7 h	8.95 (1.89)	0.04	0.21	0.21	0.01	0.832	**----**	**----**	**-----**	**-----**	**-----**
7 to 9 h	8.71 (1.87)	0.20	0.20	−0.99	0.05	0.324	**-----**	**----**	**-----**	**-----**	**-----**
More than 9 h	8.91 (1.84)	Ref.									
Smoking status											
Yes	9.31 (1.67)	0.52	0.30	1.72	0.04	0.086	**----**	**----**	**-----**	**-----**	**-----**
No	8.78 (1.88)	Ref.									
Alcohol intake											
Yes	9.32 (1.72)	0.53	0.35	1.49	0.04	0.138	**----**	**----**	**-----**	**-----**	**-----**
No	8.79 (1.72)	Ref.									
Social media usage in a day											
Less than 2 h	8.83 (1.95)	0.07	0.16	0.45	0.01	0.651	**----**	**----**	**-----**	**-----**	**-----**
2 to 5 h	8.81 (1.85)	0.05	0.14	0.40	0.01	0.690	**-----**	**----**	**-----**	**-----**	**-----**
More than 5 h	8.75 (1.88)	Ref.									
Menstruation starting age											
8 to 11 years of age	8.78 (1.96)	Ref.									
12 to 14 years of age	8.78 (1.86)	−0.01	0.14	−0.02	<−0.01	0.983	**----**	**----**	**-----**	**-----**	**-----**
More than 14 years of age	9.06 (1.86)	0.27	0.22	1.23	0.04	0.219	**-----**	**----**	**-----**	**-----**	**-----**
Type of menstruation											
Regular	8.77 (1.90)	Ref.									
Irregular	8.91 (1.81)	0.14	0.12	1.10	0.03	0.272	**-----**	**-----**	**-----**	**-----**	**------**
Duration of menstruation											
Less than 3 days	8.88 (1.98)	0.36	0.21	1.68	0.06	0.093	0.35	0.21	1.66	0.06	0.097
3 to 6 days	8.84 (1.86)	0.32	0.15	2.07	0.07	**0.039**	0.33	0.15	2.15	0.07	**0.032**
More than 6 days	8.51 (1.84)	Ref.					Ref.				

Note: B = unstandardized regression coefficient; SE = Standard error; *β* = standardized regression coefficient; Bold indicates significant (*p* < 0.05).

## 4 Discussion

MH management difficulties for women in low-income countries have earned global attention as a public health issue in recent years (12, 21). Bangladeshi women during their early reproductive years are exposed to significant risks of compromised menstrual health, primarily stemming from factors such as limited awareness, suboptimal MH practices, inadequate MH knowledge, and the presence of social stigma (3, 22). Therefore, the current study evaluated the knowledge, attitudes, and practices of early reproductive women in Bangladesh concerning mental health.

Based on the results presented, it can be inferred that the participants in this study had relatively good knowledge, positive attitudes, and appropriate practices regarding menstruation and MH. The predictors for higher knowledge scores were having education at the university level, being a student, living with a nuclear/small family, having daily sleeping time of less than 7 h, and not taking alcohol. For attitudes, the predictors were having age between 18 to 24 years, being a student, living with a nuclear/small family, not taking alcohol, and having regular type of menstruation. For practices, the predictors were having age between 25 to 35 years, having education at the university level, living in an urban area, living with a large family, and having a duration of menstruation between 3 to 6 days.

### 4.1 Comparison with other studies

The study’s findings were compared to previous research on knowledge, attitudes, and practices regarding MH among different study populations. Several studies have investigated the predictors of knowledge and attitudes toward menstruation and MH in different populations, and some of these studies have reported similar findings to the current study (20, 23).

Regarding education, a study conducted among female high school students in Nigeria found that those with higher levels of education had better knowledge and attitudes toward menstruation and MH practices (24). Similarly, a study among female university students in Malaysia reported that those with higher education levels had better knowledge and more positive attitudes toward MH (25). This is likely attributed to the awareness and knowledge fostered by higher education. Previous studies have indicated that education plays a significant role in promoting better understanding of menstruation and mental health (26, 27).

The finding that being a student is a positive predictor for both knowledge and attitudes toward menstruation and MH is consistent with previous studies. A study conducted among urban women in India found that being a student was significantly associated with better knowledge and attitudes toward MH practices (28). Another study among female high school students in Nepal reported that students had better knowledge and more positive attitudes toward MH than non-students (20). The new Bangladeshi curriculum offers improved access to comprehensive sexual and reproductive health education, particularly benefiting students who now receive information about proper menstrual hygiene practices (29).

The current study also found that living with a nuclear/small family was a positive predictor of both knowledge and attitudes toward menstruation and MH. This finding is consistent with a study among adolescent girls in Ghana, which reported that girls from nuclear families had better knowledge and more positive attitudes toward MH than those from extended families (30) because of the focused attention and education they receive within smaller family units, which allows for better guidance and awareness-building on this subject (31).

Our study found sleep status less than 7 h is a positive predicting factor of MH related knowledge. Excessive sleep or disrupted sleep patterns can hinder cognitive functioning and concentration, potentially leading to reduced attention to menstrual hygiene education and challenges in establishing consistent hygiene habits due to disrupted daily routine (32, 33). Further study is needed for more clarification.

The finding that not taking alcohol is a positive predictor of both knowledge and attitudes toward menstruation and MH. Excessive alcohol consumption can impair cognitive functions and decision-making abilities, potentially leading to misinformation or neglect of proper menstrual hygiene practices, while also diminishing the importance placed on menstrual health and hygiene due to its effects on mood and judgment (34, 35).

In terms of attitudes, the finding that having age between 18 to 24 years is a positive predictor is consistent with a study among women at Delhi in India, which reported that younger women had more positive attitudes toward MH than older (28). The finding that having regular type of menstruation is a positive predictor of attitudes toward menstruation and MH is consistent with a study among girls in India, which reported that those with regular menstrual cycles had more positive attitudes toward MH (36).

The finding that residing in a nuclear or small family positively predicts favorable attitudes toward menstruation and menstrual hygiene is intriguing. This could be attributed to the possibility that women in smaller families have increased opportunities for discussing menstruation and menstrual hygiene with family members, resulting in higher levels of knowledge and comprehension (37). Opposite result was found in a study conducted among adolescent girls of a urban slum in Assam, India (38). Overall, these findings suggest that there are certain demographic and behavioral factors that may influence attitudes toward menstruation and MH, and that interventions targeting these factors may be effective in improving attitudes and promoting menstrual health.

Regarding age, some studies have found that younger women tend to have better MH practices compared to older women, while others have found the opposite. For instance, a study conducted in India found that younger women were more likely to use sanitary pads, dispose of them properly, and wash their genitals during menstruation compared to older women (39). On the other hand, a study conducted in Korea found no association between age and MH practices (40). Younger generations tend to have increased access to comprehensive sexual and reproductive health education by new Bangladeshi curriculum, which includes information on proper MH practices (29).

Regarding education level, the findings of this study are consistent with previous research that found higher education to be associated with better MH practices (5). It is quite usual that higher education teaches us to be clean and healthy. The finding that living in an urban area is a positive predictor of appropriate practices is consistent with some studies that have found that urban women tend to have better MH practices compared to rural women (4).

According to our study living with a large family is a positive predictor of appropriate practices is in line with an Indian study (38). Because in Bangladeshi culture grandparents have a funny and interesting relationship with their grandchildren. Usually they teach grandchildren abut MH practices and sex education (41). It contradicts with an Ethiopian study that have found that living in smaller families is associated with better MH practices (15). Moreover, A Bangladeshi study found no association between good MH practices and family size (42).

According to our findings, Mother, Friends, Books/articles, Television, teacher, and social media were mentioned to be used as a common source of obtaining information about Menstruation and MH. Mother was reported as the most frequently used source of gaining knowledge regarding menstruation which is consistent with an earlier study from Bangladesh (42). Prior study related to our finding which depicted that people prefer having MH related information from close one like mother and best friends but they usually do not like to share these incidences in social media (Facebook, WhatsApp, etc.) (43, 44).

Overall, the study reveals that early reproductive-aged women in Bangladesh generally possess good knowledge, positive attitudes, and appropriate practices regarding menstrual hygiene. Factors such as higher education, student status, living in nuclear/small families, adequate sleep, and not consuming alcohol are associated with better knowledge and attitudes. This information can guide policymakers and planners in developing targeted interventions to further improve menstrual hygiene practices and promote women’s reproductive health, aligning with sustainable development goals related to water and sanitation, health, and gender equality.

### 4.2 Limitations

The study has several limitations that should be acknowledged. Firstly, the use of convenience sampling may introduce selection bias and limit the generalizability of the findings to the broader population. Secondly, collected data can be subject to recall bias/response and social desirability bias, potentially affecting the accuracy of responses because of self-reported measures. Additionally, the cross-sectional design of the study prevents establishing causal relationships and understanding changes over time. A longitudinal or prospective study will be helpful in this context. Moreover, the lack of a comparison group and limited generalizability to other regions or countries restricts the applicability of the findings. It is important to consider these limitations when interpreting the results and drawing conclusions.

## 5 Conclusion

In conclusion, this study highlights the need for targeted interventions that address the gaps in knowledge and practices related to MH among women. Efforts to improve MH practices should focus not only on educating women but also on addressing the social and environmental factors that influence these practices. Such interventions could include school-based education programs, community-based awareness campaigns, and policies that promote access to affordable MH products.

## Data availability statement

The original contributions presented in the study are included in the article/Supplementary material, further inquiries can be directed to the corresponding author.

## Ethics statement

The survey was carried out in accordance with the Helsinki Declaration of 1975. The Ethics Review Committee of the Faculty of Biological Science and Technology, Jessore University of Science and Technology, Jessore-7408, Bangladesh examined and approved the study protocol [Ref: ERC/FB ST/JUST/2022-l -0l]. All respondents were informed of the study’s goal, procedure, and ability to withdraw their data. Prior to completing the study, each participant provided informed written consent. Participants were advised that all of their information would be kept anonymous and confidential.

## Author contributions

AS, MM, and MA: conceptualization. AS, SD, MM, and MA: methodology and resources. AS and SD: data curation, formal analysis, and investigation. AS, SD, AA, SM, MH, and AP: writing – original draft preparation. MM and MA: writing – review and editing and supervision. All authors contributed to the article and approved the submitted version.
